# Illuminating gillnets to save seabirds and the potential for multi-taxa bycatch mitigation

**DOI:** 10.1098/rsos.180254

**Published:** 2018-07-11

**Authors:** Jeffrey C. Mangel, John Wang, Joanna Alfaro-Shigueto, Sergio Pingo, Astrid Jimenez, Felipe Carvalho, Yonat Swimmer, Brendan J. Godley

**Affiliations:** 1 ProDelphinus, Jose Galvez 780-E, Miraflores, Lima 18, Peru; 2 Centre for Ecology and Conservation, University of Exeter, Penryn, Cornwall TR10 9FE, UK; 3 NOAA, National Marine Fisheries Service, Pacific Islands Fisheries Science Center, Honolulu, HI 96818, USA; 4 Facultad de Biologia Marina, Universidad Cientifica del Sur, Panamericana Sur Km 19, Villa, Lima, Peru

**Keywords:** seabirds, bycatch, gillnets, vision, small-scale fisheries

## Abstract

Bycatch in net fisheries is recognized as a major source of mortality for many marine species, including seabirds. Few mitigation solutions, however, have been identified. We assessed the effectiveness of illuminating fishing nets with green light emitting diodes (LEDs) to reduce the incidental capture of seabirds. Experiments were conducted in the demersal, set gillnet fishery of Constante, Peru and compared 114 pairs of control and illuminated nets. We observed captures of a total of 45 guanay cormorants (*Phalacrocorax bougainvillii*), with 39 caught in control nets and six caught in illuminated nets. Seabird bycatch in terms of catch-per-unit-effort was significantly (*p* < 0.05) higher in control nets than in illuminated nets, representing an 85.1% decline in the cormorant bycatch rate. This study, showing that net illumination reduces seabird bycatch and previous studies showing reductions in sea turtle bycatch without reducing target catch, indicates that net illumination can be an effective multi-taxa bycatch mitigation technique. This finding has broad implications for bycatch mitigation in net fisheries given LED technology's relatively low cost, the global ubiquity of net fisheries and the current paucity of bycatch mitigation solutions.

## 1. Introduction

Gillnet bycatch is a major source of mortality in many species of seabirds, sea turtles, and marine mammals, many of which have declining populations [1–3]. Zydelis *et al*. [4], for example, estimated that seabird bycatch in gillnet fisheries likely exceeds 400 000 birds annually. Despite this threat, few mitigation solutions for seabird bycatch have been identified, let alone implemented on a large scale.

One approach to bycatch mitigation solutions is to use sensory cues to evoke behavioural changes in animals that reduce their vulnerability to fishing gear [5,6]. Such approaches have been discussed with regard to incidental captures of sea turtles [5] and elasmobranchs [7]. In addition, a recent assessment of seabird bycatch from a sensory biology perspective highlighted the importance of visual cues to seabirds and their potential use in reducing bycatch [6]. While solutions to seabird bycatch in net fisheries have been elusive, Melvin *et al*. [8] showed that the entanglement of mures and auklets in salmon drift gillnet fisheries decreased when high-visibility net mesh were used. Similarly, Trippel *et al*. [9] found that nylon barium sulfate gillnets reduced bycatch of both porpoises and seabirds and posited that the decline in seabird interactions was a result of increased net visibility.

A simple strategy of placing light emitting diodes (LEDs) on nets to create a visual alert has been shown to reduce the bycatch of sea turtles while not impacting target catch [10–12]. Such a visual cue may also be useful in mitigating seabird bycatch. The purpose of this study was to assess the effectiveness of this net illumination strategy to reduce the bycatch of seabirds in a demersal set gillnet fishery in coastal Peru.

## 2. Material and methods

Fishing trials were conducted from January 2011 to July 2013 in Sechura Bay, northern Peru. Volunteer fishers and their small-scale demersal set gillnet fishing vessels from the port of Constante accompanied by an onboard observer were used in the experiments. The primary target species were flounders (*Paralichtys* spp.), guitarfish (*Rhinobatos planiceps*) and rays (superorder Batoidea). Each fishing trial consisted of a pair of bottom set gillnets each approximately 600 m in length, composed of 24 cm stretched diagonal mesh and made of multifilament nylon. The gillnet pairs consisted of two net types: a non-illuminated control net set at a minimum of 200 m from an illuminated net that had green LEDs placed every 10 m along the float line. Nets were deployed in the late afternoon and retrieved the following morning. For each gillnet, onboard observers monitored the fishing operation, gear characteristics as well as counts of target catch and bycatch. Additional details of the experimental design are provided in Ortiz *et al*. [11], which focused on net illumination effects on target catch and sea turtle bycatch in the same fishing sets described here. We calculated seabird catch-per-unit-effort (CPUE) for each net as the number of individual seabirds captured/([net length/1000 m] × [net soak time/24 h]). As in Wang *et al*. [12], we compared seabird CPUE between the control and illuminated nets using a Wilcoxon matched-pairs signed-rank test (GraphPad Prism).

## 3. Results

**Figure 1.**
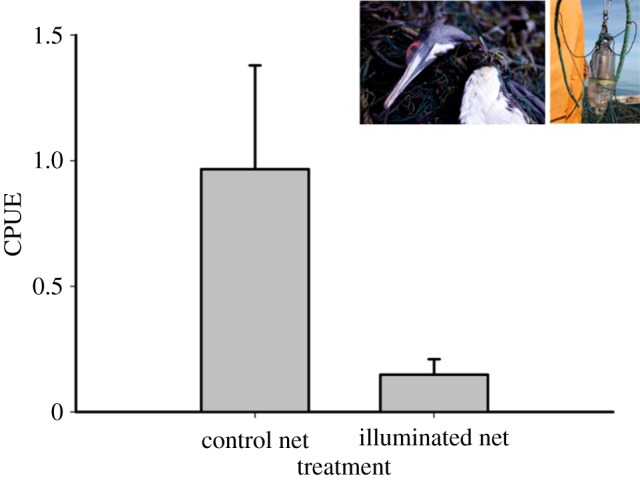
Comparison of the CPUE (no. caught per 1000 m × 24 h) of guanay cormorants between control and illuminated nets showing an 85.1% decline in illuminated nets. Error bars represent s.e. Pictured are an entangled guanay cormorant and an LED attached to a gillnet.

We deployed 114 paired nets and observed the bycatch of 45 guanay cormorants (*Phalacrocorax bougainvillii*), with 39 caught in control nets and six caught in illuminated nets (table 1). Four Peruvian boobies (*Sula variegata*) were also caught in the illuminated net but were not included in the analysis due to the small sample size. Cormorant bycatch CPUE was significantly (*p* < 0.05) higher in control nets (0.97 ± 0.41 s.e.) than in illuminated nets (0.15 ± 0.06 s.e.), representing an 85.1% decline in the cormorant catch rate (figure 1). All 45 cormorants were recovered dead. As detailed in Ortiz *et al*. [11], the predicted mean CPUE of the target catch of guitarfish, rays and flounders in these same illuminated sets was unchanged compared to the control nets.

**Table 1.** Summary of total fishing effort and guanay cormorant bycatch by net type (control and illuminated) for paired gillnet sets in Sechura Bay, Peru.

net type	sets	total effort (km × 24 h)	guanay cormorant bycatch
control	114	48.96	39
illuminated	114	47.71	6

## 4. Discussion

In the previous study of this active small-scale fishery, we showed that net illumination reduced green turtle (*Chelonia mydas*) bycatch by 63.9% and had no impact on catch rates of the three target species of flounder, guitarfish, rays and flounders [11]. For this study, we show that net illumination also reduced the bycatch of cormorants by 85.1% in these same fishing sets (table 1 and figure 1).

The potential application of net illumination at reducing bycatch of multiple taxa (i.e. seabirds and sea turtles) in net fisheries is promising for several reasons. First, in many cases, bycatch of these taxa co-occurs [4]. Second, identification of one bycatch mitigation technology that addresses multiple taxa could help encourage and streamline adoption and reduce implementation costs (i.e. as opposed to having to implement several taxa-specific mitigation measures). Third, the global ubiquity of net fisheries means that there is potential for large conservation gains if net illumination proves similarly successful in other regions or fisheries. This is particularly true regarding small-scale fisheries, for which the identification of relatively low-cost, easy to implement solutions that do not impact target catch may be especially important towards promoting implementation.

While several studies show the effectiveness of net illumination in reducing sea turtle interactions with gillnets [10–12], this is the first to show that net illumination also reduces seabird bycatch. As with sea turtles, the specific mechanism by which net illumination reduced seabird interactions is unclear. In this study, seabird bycatch was composed primarily of guanay cormorants which forage by pursuit underwater [13]. Net illumination could simply have increased the visual signature of the gillnets and allowed the seabirds to better avoid becoming entangled [6,8,9]. Tests of net illumination in fisheries with other seabird assemblages (e.g. penguins and procellariformes), or that use different foraging strategies could help explain the specific mechanism of this bycatch reduction while also determining if net illumination could be broadly effective at reducing seabird bycatch [14]. Seabird species at high risk globally from bycatch in net fisheries include ducks, loons and auks, all of which dive while foraging [4,6]. Additional tests of net illumination in fishing areas with plunge diving species would be particularly useful given the observed bycatch in this study of four Peruvian boobies in the illuminated nets.

Development of net illumination as a bycatch reduction technology should continue to focus on other bycatch taxa such as elasmobranches and marine mammals. In addition, differences in the behavioural response of fish species may lead to beneficial changes in target catch rates. If so, it may be possible to match specific wavelengths of net illumination to the specific conservation needs of a particular fishery. Other research directions could include assessments of LED intensities, wavelengths and spacing along the net, as well as comparisons with other bycatch reduction techniques, including those that use other sensory strategies (e.g. high-visibility net panels, acoustic alarms) [6,15]. Tests under true fishery conditions could be particularly important in assessing net illumination's effectiveness and encouraging its uptake by fishers.

Here, we have described a technology that has been shown to reduce bycatch of two marine taxa of global conservation concern [11], and to do so with no impact on the fishery's target catch rate [11]. These findings alone are encouraging. In addition, these results are with fisheries that are massive in scale and extent, and employs a technology that could potentially become cost-effective even in small-scale fisheries [11], increasing the urgency for further testing leading to broader implementation. Ultimately, such technologies could help increase opportunities for fisheries to be sustainable for both target and bycatch species.

## Supplementary Material

Supporting dataset

## Data accessibility

The dataset supporting this article has been uploaded as electronic supplementary material.
